# Evaluation of experimental genetic management in reintroduced bighorn sheep

**DOI:** 10.1002/ece3.97

**Published:** 2012-02

**Authors:** Zachary H Olson, Donald G Whittaker, Olin E Rhodes

**Affiliations:** 1 Department of Forestry & Natural Resources195 Marstellar StreetPurdue UniversityWest Lafayette, Indiana 47907; 2 Oregon Department of Fish and Wildlife3406 Cherry Avenue NE, Salem, Oregon 97303

**Keywords:** Bighorn sheep, genetic management, genetic rescue, inbreeding depression, outbreeding depression, *Ovis canadensis californiana*

## Abstract

Positive demographic responses have been reported in several species where the immigration or supplementation of genetically distinct individuals into wild populations has resulted in a genetic rescue effect. However, rarely have researchers incorporated what could be considerable risk of outbreeding depression into planning for genetic management programs. We assess the genetic effects of an experiment in genetic management involving replicate populations of California bighorn sheep (*Ovis canadensis californiana*) in Oregon, USA, which previously experienced poor productivity and numerical declines. In the experiment, two declining populations were supplemented with ewes from a more genetically diverse population of California bighorn sheep in Nevada. We incorporated analysis of genetic samples representing both experimental populations prior to supplementation, samples from the supplemented individuals, and samples collected from both experimental populations approximately one generation after supplementation. We used genetic analyses to assess the integration of supplemented and resident populations by identifying interpopulation hybrids. Further, we incorporated demographic simulations to assess the risk of outbreeding depression as a result of the experimental augmentation. Finally, we used data from microsatellites and mitochondrial sequences to determine if genetic management increased genetic diversity in the experimental populations. Our analyses demonstrated the success of genetic management by documenting interpopulation hybrids, identifying no evidence for outbreeding depression as a result of contact between the genetically distinct supplemented and resident populations, and by identifying increased population-level metrics of genetic diversity in postsupplementation populations compared with presupplementation levels.

## Introduction

An infusions of genetic diversity that results in reduced evidence of inbreeding depression, as measured by various demographic parameters (e.g., fecundity or juvenile survival), has been termed “genetic rescue” (Thrall et al. 1998), and the responses of wild populations to cases of natural (Vilà et al. 2003) and planned (Westemeier et al. 1998; Madsen et al. 1999) genetic rescue have been striking. However, purposefully managing a population to produce a genetic rescue effect is a complex undertaking, the success of which depends upon knowledge of the genetic architecture of the population that is not always available to the manager (Tallmon et al. 2004). For example, a rescue effect in populations exhibiting inbreeding-like reductions in fitness due to drift (see Leberg and Firmin 2008) most likely occurs because the influx of genetic diversity increases heterozygosity in hybrid (i.e., admixed) individuals. In such cases increased heterozygosity enhances fitness of hybrids via overdominance (i.e., a heterozygote advantage) or by masking the mildly deleterious alleles responsible for decreased fitness (Edmands 1999; Keller and Waller 2002; Charlesworth and Willis 2009). Alternatively, if introgression of immigrant genes disrupts positive gene–environment interactions, or results in a breakdown of favorable epistatic interactions, that same population may exhibit reduced hybrid fitness (Lynch 1991; Edmands 1999; Burke and Arnold 2001): termed outbreeding depression. Despite the risk of outbreeding depression, many extant populations, particularly those established via reintroduction efforts, contain too few individuals to resist reductions in fitness due to drift and require genetic management to maintain population persistence and evolutionary potential (Frankham 2005).

In natural metapopulations, interpopulation migrants also may serve to buffer populations from the deleterious effects of inbreeding depression (Nieminen et al. 2001; Haag et al. 2002), and thus, for species exhibiting metapopulation characteristics, the negative effects of population isolation (Lande 1995) may be remediated through immigration. For example, bighorn sheep (*Ovis canadensis*), which once existed throughout their range in natural metapopulations characterized by the patchy distribution of suitably rugged habitat (Bleich et al. 1996), may have overcome the negative effects of population isolation through periodic migration of individuals. This premise is supported by evidence on two fronts: remaining metapopulations exhibit relatively common extinctions of and movement between populations (Schwartz et al. 1986; Berger 1990; Bleich et al. 1996), and recent evidence indicates that bighorn sheep exhibit no behavioral avoidance of inbreeding in the wild (Rioux–Paquette et al. 2010). Unfortunately, the genetic and demographic benefits associated with migrants are unavailable to many extant bighorn sheep populations because reintroduction efforts have yet to establish networks of populations within dispersal range or because traditional migration corridors have been bisected by human development (Risenhoover et al. 1988; Epps et al. 2005). As a result of actual or effective isolation of these bighorn populations, and despite the overwhelming success of sheep reintroduction efforts in general (Valdez and Krausman 1999), many reintroductions fail (Risenhoover et al. 1988) and some reintroduced populations have experienced decreased productivity possibly as a result of genetic drift and/or inbreeding depression.

Research initiated byWhittaker et al. (2004) provides an example of the concerns associated with the isolation and demographic declines of reintroduced bighorn sheep populations. In this research,Whittaker et al. (2004) investigated two reintroduced populations of California bighorn sheep in Oregon, Steens Mountain (SM) and Leslie Gulch (LG), which experienced decreased lamb-to-ewe ratios indicative of decreased herd productivity in the mid 1990s. With no evidence that disease was the cause for concurrent numerical declines, the Oregon Department of Fish and Wildlife (ODFW) investigated the extent of inbreeding in the populations as a potential source for the observed declines in productivity (Whittaker et al. 2004). To accomplish this, ODFW measured genetic diversity parameters for five reintroduced populations in Oregon (Aldrich Mountain, Hart Mountain, LG, Lower John Day, and SM) in 2000 and 2001 and compared these values to those obtained for the Santa Rosa Mountains (SR) population in Nevada. The Santa Rosa population was chosen for comparison because it was reintroduced using different source stock than the populations reintroduced in Oregon and was considered an example of a demographically and numerically healthy bighorn sheep population (Whittaker et al. 2004). Whereas genetic diversity parameters for the Santa Rosa population were well within the normal range observed for this species, genetic diversity estimates of the California bighorn sheep populations examined from Oregon were among the lowest ever reported in any *Ovis* population (Whittaker et al. 2004; Hogg et al. 2006).

Thus, in an effort to increase genetic diversity, improve herd productivity, and determine the efficacy of further genetic management for reintroduced bighorn sheep in Oregon, experimental supplementations were conducted in SM and LG in 2000 and 2001, respectively (Whittaker et al. 2004). The source population for these supplementations was the Santa Rosa California bighorn sheep population that exhibited higher levels of genetic diversity than either the SM or LG populations (Whittaker et al. 2004). Utilizing this quasi-experimental framework, our goal in this research was to test hypotheses indicative of success for this trial in genetic management using data from the treatment populations gathered approximately one-generation postsupplementation. Specifically, we used genetic methods combined and demographic models to (1) detect interpopulation hybrids (i.e., hybrids between Santa Rosa and each of the Oregon herds), (2) test for a signature of outbreeding depression by investigating if lamb-survival rates for interpopulation hybrids were less than those of resident populations prior to supplementation, and (3) evaluate whether measures of genetic diversity increased or levels of relatedness decreased in treatment populations after supplementation.

## Methods

### Study areas

SM is located in Harney County of southeastern Oregon. Bighorn sheep primarily inhabit the east face of SM, which rises a vertical mile from the surrounding landscape (Whittaker et al. 2004). The population was reintroduced via transplants of four and seven California bighorn sheep in 1960 and 1961, respectively, from Hart Mountain (Coggins et al. 1996). Hart Mountain itself was the first herd of California bighorn sheep reintroduced to Oregon, and was established using sheep transplanted from Williams Lake, British Columbia, in 1954 (Coggins et al. 1996). The SM population grew large enough in number by 1985 to permit its use as a source herd for further reintroductions and supplementations in Oregon (ODFW 2003). However, SM experienced numerical declines of 30% from the late 1980s through the 1990s along with generally low lamb recruitment from 1995 to 1999: lambs per 100 ewe estimates (i.e., a proxy for lamb survival) from this period ranged from 11 to 26 (Whittaker et al. 2004). In January of 2000, as part of a genetic management experiment, 15 California bighorn ewes were translocated to SM from the SR of Nevada (Whittaker et al. 2004). It was assumed that supplemented ewes ≥2 years old (*N* = 13) likely were pregnant when released.

LG is a rocky gorge that branches from the Lower Owyhee River canyon in Malheur County of eastern Oregon (Whittaker et al. 2004). California bighorn sheep were reintroduced to LG with a transplant of 17 sheep from Hart Mountain in 1965 (Coggins et al. 1996). Similar in history to SM, around 1985, the LG population grew large enough to support translocations of California bighorn sheep for restoration efforts in Oregon (ODFW 2003). Also similar to SM, LG experienced numerical declines of over 30% during the 1990s with generally low lamb recruitment (lambs: 100 ewes ranged from 7 to 24 during 1995–1999; details inWhittaker et al. 2004). As the replicate population in this genetic management experiment, LG received 16 California bighorn ewes translocated from SR of Nevada in January of 2001 (Whittaker et al. 2004). Of these ewes, 12 likely were pregnant when released based on their age (i.e., ≥2 years old). Only ewes were used in supplementation cohorts because female bighorn sheep breed more consistently than males, and it was thought that this would aid the process of introgression.

The source population used for both experimental supplementations was the SR herd of northeastern Nevada (Whittaker et al. 2004). Sheep were reintroduced to SR using transplants from two different source populations between 1978 and 1998 and the population exhibited higher genetic diversity than any of Oregon's extant California bighorn sheep populations (Whittaker et al. 2004).

### Sample collection

**Figure 1:**
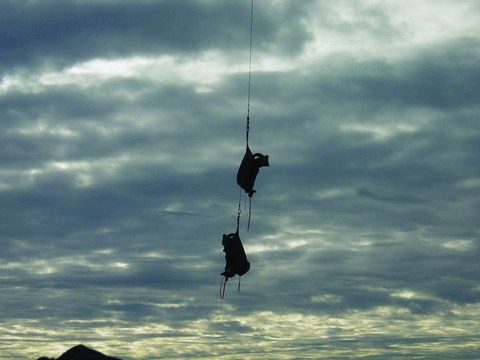
Study area locations: SM = Steens Mountain, LG = Leslie Gulch, and SR = Santa Rosa Mountains. SM and LG were subjected to experimental genetic management in 2000 and 2001, respectively, during which they were supplemented with ewes from the more genetically diverse SR population.

We obtained genetic samples from California bighorn sheep originating from SM, LG, and SR. ODFW provided genomic DNA from samples used inWhittaker et al. (2004): 41 samples from the experimental populations SM and LG prior to supplementation and 31 samples comprising the supplemented individuals from SR (*n* = 72 total). We collected additional tissue samples opportunistically from hunter-killed sheep. SM and LG were resampled in 2006 (SMP, “P” stands for postsupplementation; *n* = 48) and 2007 (LGP; *n* = 50), approximately one generation following experimental genetic management to track the progress of introgression. In both postsupplementation sampling events (Fig. 1), the helicopter capture-crew (Leading Edge Aviation, LLC, Lewiston, ID) focused their efforts on individuals ≤6 years old from the full range of the herd to enhance the likelihood that sampled individuals were a random subset of those conceived after the supplementations of 2000 (SM) and 2001 (LG). Because aging sheep (ewes in particular) from a helicopter is challenging, we expected some captures to be >6 years old once their age could be determined more accurately in hand. We redacted individuals >6 years old from the sample during subsequent analyses. Muscle tissue was collected from hunter-killed sheep whereas live-captured sheep were sampled using a 0.95-cm diameter ear punch (i.e., 0.375-in; Stone Manufacturing and Supply Co., Kansas City, MO). Experienced researchers recorded age (determined by counting horn growth annuli) and sex of sampled individuals. Tissue samples were stored in 2-mL, screw-top vials filled with 95% ethanol or silica desiccant beads. All samples were shipped to the genetics lab at Purdue University and stored at –80°C until DNA extraction.

### Laboratory methods

Genomic DNA was extracted from tissue samples using a modified ammonium acetate protocol (Gentra Puregene kit; Qiagen Inc., Valencia, CA) and rehydrated in 100-µl TLE (10 mM Tris-HCL, 0.1 mM EDTA). We assessed extraction success by visualizing the product of gel electrophoresis on a Gel Doc XR system (Bio-Rad, Hercules, CA). DNA concentrations were quantified in successful extractions using a NanoDrop 8000 spectrophotometer (Thermo Scientific, Waltham, MA). We diluted working stocks of genomic DNA to 20 ng/µl and stored the original extractions at –80° C to slow the degradation of extracted DNA.

#### Microsatellites

We used polymerase chain reaction (PCR) to amplify 23 microsatellite loci from each sample, including the 11 markers used byWhittaker et al. (2004). These loci were chosen from a suite of 56 used in previous mountain sheep research (Olson et al. 2008; Appendix 2). We amplified each locus separately in 10-µl reaction volumes that contained 20 ng DNA template, 0.25 µM of each primer, 0.2 mM of each dNTP, an optimized quantity of MgCl_2_ (Table S1), 1× reaction buffer (10 mM Tris-HCL, 50 mM KCL, 0.05 mg/µl BSA), and 1 unit of *Taq* DNA polymerase. We used the following thermocycler profile for all loci: 94°C for 2 min; 30 or 35 cycles of 94°C for 30 sec, optimized annealing temperature for 30 sec (Table S1), and 72°C for 30 sec; then 72°C for 10 min and a final extension at 60°C for 45 min. Successful amplification was verified by electrophoresis of PCR products (we included the PCR positive and negative controls from each reaction) through 2% agarose gel stained with ethidium bromide. Amplified fragments were electrophoresed using an ABI 3730xl automated sequencer (Applied Biosystems, Carlsbad, CA). We assigned genotypes after importing the electrophoretic data into GeneMapper version 3.7 (Applied Biosystems). We employed the standard quality control measures for microsatellite datasets in our lab (described inDrauch et al. 2008). Briefly, (1) we included allelic standards for each locus in each run on the sequencer, (2) an experienced researcher independently rescored ∼20% of the locus per sample combinations to ensure repeatability of scoring, (3) all ambiguous or low-quality genotypes (signal strength <100 in GeneMapper) were reamplified to confirm the genotype, and (4) we reamplified and scored a randomly selected ∼20% of the dataset to assess genotyping error rates.

#### Mitochondrial DNA

We amplified a 515 base pair fragment of the mitochondrial control region using the following PCR conditions: an initial denature step of 5 min at 94°C, 35 cycles of 94°C for 60 sec, 65°C for 70 sec, and 72°C for 90 sec, followed by a final 72°C extension step of 5 min. Each 10-µl reaction volume contained 20 ng template DNA, 0.25 µM forward and reverse primer (Table S1), 0.2 mM of each dNTP, 1.8 mM MgCl_2_, 1× reaction buffer (10 mM Tris-HCL, 50 mM KCL, 0.05 mg/µl BSA), and 1 unit of *Taq* DNA polymerase. Amplification success was verified via electrophoresis as above. PCR products were cleaned by precipitating the DNA with a sodium acetate solution (0.12 mM NaOAc in 100% ethanol), centrifuging to form a pellet, washing the pellet with 70% ethanol, and resuspending the PCR product in water. We then cycle-sequenced approximately 10 ng of cleaned PCR product in 10 µl reaction volumes containing 5 pmol forward or reverse primer, 1 µl Big Dye Terminator version 3.1 (Applied Biosystems), and 3 µl of 5× buffer (Applied Biosystems). We used the following sequencing-reaction profile: 98°C for 5 min, followed by 60 cycles of 98°C for 30 sec, 50°C for 15 sec, and 60°C for 2 min. Sequencing products were cleaned using the same sodium acetate protocol as above prior to being rehydrated in 30 µl sterile water. Cleaned sequencing products were run on an ABI 3730xl automated DNA sequencer (Applied Biosystems). We aligned and manually edited the resulting sequences using Sequencher version 4.1 (GeneCodes Corp., Ann Arbor, MI). To ensure the quality of our dataset, all samples were sequenced in forward and reverse directions.

### Data analysis

For microsatellite data, we used program create version 1.33 (Coombs et al. 2008) to facilitate data conversion between genetic data analysis programs. We tested for locus-specific deviations from Hardy–Weinberg equilibrium (HWE) using Fisher's exact tests and for pairwise deviations among loci from linkage equilibrium in genepop version 4.0 (100,000 steps in the Markov chain; 100 batches with 1000 iterations; Raymond and Rousset 1995). For mitochondrial data, we collapsed sequences into haplotypes and enumerated variable sites among haplotypes using program fabox version 1.35 (Villesen 2007). We then inferred haplotype genealogies using the method of maximum parsimony as implemented in program tcs version 1.21 (Clement et al. 2000). We identified all haplotypes unique to SR prior to supplementation for use in diagnosing the extent of introgression between resident and supplemented lineages.

#### Documenting introgression

We measured the extent of introgression (i.e., the number of hybrids) between resident populations (SM and LG) and the supplemented SR individuals using information from both mitochondrial and nuclear DNA. First, we identified all individuals in postsupplementation samples (SMP and LGP) that contained mitochondrial haplotypes unique to SR prior to supplementation. Since only ewes were supplemented from SR, unique-SR haplotypes could only have occurred in postsupplementation sampling if individuals were part of a maternal lineage tracing back to a supplemented ewe. We assumed the assignment of mitochondrial haplotypes to individuals was error free.

Second, we used the Bayesian clustering approach implemented in program newhybrids version 1.1 (Anderson and Thompson 2002) to assign individuals to categories of descent based on their multilocus microsatellite genotypes. newhybrids uses Markov chain Monte Carlo sampling to compute the posterior probabilities that each individual belongs to a particular category of descent based on allele frequencies provided for two parent populations. In this study, the “parent” populations were the resident herd (SM or LG) and the actual supplemented individuals from SR. Categories of descent were pure-resident, pure-supplemented, F1 (i.e., pure resident × pure supplemented), F2 (i.e., F1 × F1), and two backcrosses: F1× pure resident (BX_res_) and F1 × pure supplemented (BX_sup_). Further hybrid classes were not considered because they are progressively more difficult to diagnose using molecular markers (Anderson and Thompson 2002) and ultimately were unlikely given the duration of the study (i.e., about one generation). Beyond the specific category of descent, we considered an individual a hybrid if the sum of posterior probabilities for F1, F2, BX_res_, and BX_sup_ was > 0.50. We analyzed both datasets (SMP and LGP) many times using different, over-dispersed random seeds after verifying that the sampler was mixing properly as recommended byAnderson and Thompson (2002). We used Jeffries-like priors for both the mixing proportions and allele frequencies, although similar results were obtained in multiple analyses performed using uniform priors (Anderson and Thompson 2002). All analyses were conducted using 10^4^ burn-in sweeps followed by 10^5^ sweeps on which data were collected.

We then enumerated hybrid individuals from SMP and LGP samples based on the following criteria. We counted all individuals identified as hybrids using their multilocus microsatellite genotypes in newhybrids. To that number we added individuals that harbored an mtDNA haplotype unique to SR prior to supplementation if their multilocus genotype profile (via newhybrids) indicated that they were of pure resident descent (i.e., likely misassigned as a result of limited power to detect hybridization using the microsatellite dataset alone). Individuals similarly harboring SR haplotypes, but with multilocus genotypes assigning back to the supplemented population, also were possible because the supplemented ewes ≥2 years old probably were pregnant with pure-SR offspring at the time of their release. Thus, pure-supplemented individuals (≤6 years old) could then be detected in postsupplementation sampling if a pure-supplemented ram, carried in utero by a supplemented ewe, survived to later breed with a supplemented ewe. However, we consider this biological scenario to be unlikely.

Finally, to assess the power of our suite of microsatellite markers to identify various hybrids, we used program hybridlab version 1.0 (Nielsen et al. 2006) to simulate 1000 multilocus genotypes in each of four hybrid categories: F1, F2, and 2 backcross categories (BX_res_ = F1 × pure-resident and BX_sup_ = F1 × pure-supplemented). Two sets of simulated hybrid individuals were created using the appropriate resident (SM or LG) and supplemented (SR) genotypes as starting parent populations. We then assessed the ability of the program newhybrids to correctly identify hybrid individuals and to assign hybrid individuals to the appropriate category of descent using identical program parameters to those described above (i.e., Jeffries-like priors, 10^4^ burn-in sweeps, and 10^5^ sweeps for data collection). We then calculated the number of correct and incorrect assignments made by newhybrids for each category of simulated hybrids.

#### Diagnosing the potential for outbreeding depression

We constructed stochastic, transition-matrix population models to simulate the range of lamb survival values that could account for the observed proportion of hybrid individuals sampled from SMP and LGP one generation after supplementation. We chose to investigate lamb survival because juvenile viability is the demographic parameter most affected by inbreeding and outbreeding depression (Ralls et al. 1988; Keller and Waller 2002; Edmands 2007; Rioux–Paquette et al. 2011). Thus, if lamb survival of hybrid individuals remained unchanged or increased relative to baseline levels for the resident populations, there would be no immediate evidence of outbreeding depression resulting from interpopulation crosses.

We simulated survival and reproduction of the supplemented ewes at SM and LG in separate spreadsheet models through 6 years, which corresponded with the number of years between the actual supplementations (2000 and 2001) and postsupplementation sampling (2006 and 2007 in SM and LG, respectively). Starting conditions for each simulation were the specific ages of the supplemented ewes (different for SM and LG). We assumed the supplemented ewes ≥2 years old could be pregnant upon their arrival, but also that we could detect pure-supplemented individuals in postsupplementation sampling using molecular methods and remove them from further calculations. Inference into lamb survival rates for pure-supplemented individuals was beyond the scope of this study.

**Figure 2:**
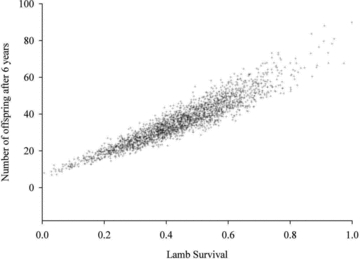
Example output from 2500 demographic simulations incorporating stochastic, age-specific survival and fecundity parameters. As lamb survival increases, the number of individuals (≤6 years old) belonging to supplemented lineages 6 years postsupplementation increases. Each simulation was initiated from a population with ages identical to the actual ewes supplemented as part of experimental genetic management; the simulations shown here utilized data from the Steens Mountain, Oregon, supplementation of 2000. Data for use in analyses were collected from runs of 100,000 simulations.

In each simulated year, all individuals alive in the model advanced to the next age class with a probability equal to their age-specific survival rate. Then, all ewes produced a single lamb (twinning is rare in mountain sheep; Lawson and Johnson 1982) with probability equal to their age-specific fecundity. We assumed the sex ratio was equal at birth (Lawson and Johnson 1982), all male offspring maintained survival probabilities identical to the females, and only females produced lambs. At the beginning of each simulation, new values of all demographic parameters were drawn at random from parameter-specific, normal distributions that we constructed based on data from long-term studies of wild mountain sheep populations (Table 1). After the random draw of parameter values, individuals in the model progressed through 6 simulated years, at the end of which we recorded the number of males and females in the model ≤6 years old and associated that number with the lamb survival value for that simulation. The number of individuals ≤6 years old after the simulation was dependent on the lamb survival rate, but also varied as a function of age-specific survival and fecundity values (Fig. 2). We ran the simulation 10^5^ times each for SM and LG to generate measures of central tendency for lamb survival at any given value of the number of individuals ≤6 years old (Appendix 1).

**Table 1:** Mean and standard deviation (SD) for age-specific survival and fecundity parameters used to simulate the expected number of hybrid individuals in postsupplementation sampling of California bighorn sheep populations in Oregon

		Survival^1^,^2^		Fecundity^2^	
			
Stage	Age	Mean	SD	Mean	SD
Lamb	1	0.450	0.1500	0	0
Yearling	2	0.825	0.0625	0.300	0.0125
Adult	3–7	0.940	0.0200	0.950	0.00625
Old adult	8–13	0.875	0.0375	0.950	0.00625
Past prime	14–16	0.600	0.1000	0.400	0.0125

1 Survival = probability of transition to the specified age. For example, lamb survival was the probability of transition to age 1.

2 Age-specific survival and fecundity distributions were based on values reported from long-term studies of bighorn sheep populations: Jorgenson et al. (1997), Bérubé et al. (1999), Loison et al. (1999), Festa–Bianchet et al. (2006), and Festa–Bianchet and King (2007).

We determined the number of individuals in the actual population that were hybrids by multiplying the proportion of hybrid individuals from postsupplementation sampling (see *diagnosing introgression* above) by the population size of SMP in 2006 and LGP in 2007. Population size estimates (calculated using POP-II; Bartholow 1995) were provided by ODFW from yearly herd-inventory data, but were estimates of total population size, not just individuals ≤6 years old. To determine how many individuals were available for sampling from SM and LG in 2006 and 2007, respectively (i.e., the number ≤6 years old), we created a Leslie matrix (Leslie 1945) using mean values of survival and fecundity (Table 1) and generated the stable age distribution via eigenanalysis in matlab version 7.10 (MathWorks, Natick, MA) followingCaswell (2001). We then calculated the adjusted population size (i.e., number of individuals available for sampling because they were ≤6 years old) by multiplying the proportion calculated from the stable age distribution by the population size estimate.

We then multiplied the observed proportion of hybrids (from *diagnosing introgression* above) by the adjusted population size to obtain the expected number of hybrid individuals in the actual SM and LG herds 6 years after supplementation. The mean ± 2 standard deviation (i.e., a pseudo-95% confidence interval) of lamb survival values from all simulations resulting in populations within ±10% of the expected number of hybrids were calculated for comparisons with observed, baseline (i.e., pregenetic management) lamb survival values in both SM and LG. We chose to incorporate this ±10% adjustment a priori to account for any unmeasured deviations from our assumptions, for example, those deviations due to variance underlying our original population size estimates or divergence from a stable age distribution.

We fully acknowledge the imprecise nature of this modeling exercise. However, our purpose was not to predict exact outcomes, but rather to expose plausible edges of the distributions of lamb survival values for hybrid individuals in SM and LG. Ideally, we would have been able to compare predicted lamb survival for supplemented ewes to that of resident ewes by modeling each group independently. Unfortunately, our models required knowledge of individual ages, data that was only available for the supplemented ewes. Acquiring ages for ewes in the resident herds would have involved helicopter capture of a large number of individuals, and would have been prohibitively expensive.

We used yearly herd-inventory estimates of lambs per 100 ewes from 1990 to 1999 obtained from ODFW as proxies for observed, baseline lamb survival in SM and LG. These values approximate lamb survival to at least 9 months of age as herd inventories are conducted during late winter through early summer. As such, these values likely overestimate true lamb survival to age 1 (i.e., due to unsampled mortality during months 9–12), and therefore provide a conservative comparison for our model-predicted lamb survival values.

#### Describing population-level effects of genetic management

We assessed the effects of experimental genetic management on several metrics of genetic diversity and on estimates of relatedness by describing changes from pre- to postsupplementation. First, we used paired *t*-tests in SAS version 9.1 (SAS Institute Inc., Cary, NC) to determine if locus-specific expected heterozygosity (Nei's unbiased H_E_; Nei 1978) calculated in genalex version 6.3 (Peakall and Smouse 2006) and allelic richness (A_R_; rarefacted to control for unequal sample sizes) calculated in program fstat version 2.9 (Goudet 1995) increased after genetic management compared to pregenetic management levels. Second, we calculated average pair-wise estimates of relatedness (*r*; Lynch & Ritland 1999) in genalex for SM, SMP, LG, and LGP. We used 9999 bootstraps to calculate a 95% confidence interval around each population mean to facilitate comparisons of relatedness between populations pre- and postsupplementation. Third, we calculated the number of mtDNA haplotypes, number of unique (i.e., private) haplotypes, haplotype diversity (H_D_; analogous to single locus heterozygosity and maximized in a sample when haplotypes occur at equal frequencies; Tajima 1983; Nei 1987), and the average number of pairwise nucleotide differences among haplotypes within populations (*k*; Tajima 1983) in program arlequin version 3.5 (Excoffier and Lischer 2010). We qualitatively compared these measures of mtDNA diversity between pre- and postsupplementation populations.

## Results

We successfully extracted genomic DNA from 171 samples (Table 2). We excluded from all analyses four microsatellite loci that failed to amplify reliably (BM1818, CELJP23, OarAE16, and TGLA94), two loci that exhibited genotyping error rates >5% (OarFCB193 and BM1225), and one locus that was monomorphic across all populations (BM4107). For the remaining 16 loci (Appendix 2) that we used in all analyses, the genotyping error rate was <2.5% per locus and <0.5% overall. The missing data rate was 0.5% overall, although locus OarFCB304 had a missing data rate of 5.8%. After correction for multiple tests, one locus/population combination exhibited significant deviation from HWE: BM4505 in SR (*F*_IS_ = 0.448, *P* < 0.05). There was no evidence for linkage disequilibrium among microsatellite loci in the populations (data not shown).

**Table 2:** Genetic diversity metrics for two populations of California bighorn sheep. Estimates were calculated using 16 polymorphic microsatellites and 515 base pairs of mitochondrial control region sequence

		Microsatellite			mtDNA								
			
Population^1^	Samples	H_O_	H_E_	A_R_	*n*	H_T_	Haplotype frequency					H_D_	*k*
									
							A	B	C	D	E		
SR	31	0.48	0.56	3.31	31	4	12	3	15	1		0.626	11.32
SM	19	0.42	0.41	2.54	13	1					13	0.000	0.000
SMP	48	0.44	0.47	3.16	48	3	8		1		39	0.318	6.871
LG	23	0.39	0.43	2.71	22	1					22	0.000	0.000
LGP	50	0.46	0.48	3.34	48	3	6		5		37	0.387	6.778
Overall	171				162	5	26	3	21	1	111		

a SR = Santa Rosa Mountains; SM = Steens Mountain; SMP = SM sampled one generation postsupplementation; LG = Leslie Gulch; LGP = LG sampled one generation postsupplementation.

H_O_ = observed heterozygosity; H_E_ = expected heterozygosity; A_R_ = allelic richness (rarefacted to accommodate unequal sample sizes); *n* = sample size; H_T_ = number of haplotypes; H_D_ = haplotype (i.e., gene) diversity; *k* = mean number of nucleotide differences among haplotypes.

We acquired forward and reverse mtDNA control region sequences from 162 of 171 samples (Table 2). We documented 28 variable sites segregating among five haplotypes: four haplotypes were unique to SR prior to the experimental supplementations (Table 2). Haplotype E was most prevalent (68.5% overall) and was the only haplotype documented in SM and LG prior to supplementation (Table 2). Based on maximum parsimony genealogies, all SR-unique haplotypes (A–D; Table 2) were between nine and 31 step mutations from haplotype E. Thus, we considered haplotypes A–D to be perfect predictors of supplemented maternal lineages in postsupplementation samples.

### Documenting introgression

Using information from mitochondrial haplotypes and analyses of multilocus microsatellite genotypes, we documented nine hybrids from SMP (Table 3). In addition, one individual in postsupplementation sampling of SMP was born prior to the supplementation based on its age at the time of capture (a ewe ≥8 years old) and three individuals represented pure-supplemented lineages based on evidence from their mtDNA and multilocus microsatellite genotypes. These individuals were excluded from further analyses. Thus, the proportion of hybrid individuals from SMP was 20.5% (9 of 44; Table 3). We documented 11 hybrids from LGP. In addition, two individuals in postsupplementation sampling were born prior to supplementation (a 7-year-old ram and a 9-year-old ewe), and one individual was pure supplemented based on mtDNA and microsatellite evidence. These individuals were excluded from further analyses. Therefore, the proportion of hybrid individuals from LGP was 23.4% (11 of 47; Table 3).

**Table 3:** Individuals identified as hybrids approximately one generation after experimental genetic management of two California bighorn sheep populations in Oregon (i.e., admixed between resident “Res.” and supplemented “Sup.” lineages) based on multilocus microsatellite genotype (program newhybrids) or the presence of a mitochondrial haplotype originating from the supplemented lineage. We present the likelihood of assignment to particular categories of hybrid descent (in parentheses), but we distinguished only between hybrid and nonhybrid for analyses

				mtDNA		newhybrids	
					
Population	Sample	Sex	Age	Haplotype	Haplotype origin	Assignment	Likelihood
Steens Mountain	06–23	Ram	3	E	Res.	Hybrid (F2)	0.64
	06–25	Ram	4	A	Sup.	Hybrid (F2)	0.61
	06–27	Ram	4	A	Sup.	Hybrid (F2)	0.65
	06–29	Ram	4	A	Sup.	Hybrid (F2)	0.53
	06–30	Ewe	4	A	Sup.	Res.	0.87
	06–31^1^	Ram	5	C	Sup.	Supp.	0.88
	06–32	Ram	3	A	Sup.	Hybrid (F2)	0.66
	06–37^1^	Ewe	2	A	Sup.	Supp.	0.97
	06–45	Ram	5	A	Sup.	Hybrid (F2)	0.53
	06–46	Ewe	2	E	Res.	Hybrid (F2)	0.58
	06–52^1^	Ram	1	A	Sup.	Supp.	0.68
	06–54	Ewe	lamb	E	Res.	Hybrid (F2)	0.65
Leslie Gulch	07–105	Ewe	3	A	Sup.	Hybrid (F1)	0.81
	07–115	Ewe	2	A	Sup.	Res.	0.99
	07–117	Ewe	4/5	A	Sup.	Hybrid (F1)	0.74
	07–121	Ewe	5	A	Sup.	Hybrid (F2)	0.52
	07–126	Ewe	4	A	Sup.	Hybrid (F1)	0.54
	07–130	Ewe	3	A	Sup.	Hybrid (F1)	0.47
	07–133	Ewe	4/5	A	Sup.	Res.	0.98
	07–134	Ewe	3	A	Sup.	Hybrid (F2)	0.64
	07–135^1^	Ram	1	A	Sup.	Supp.	0.68
	07–142	Ram	1	A	Sup.	Hybrid (F1)	0.66
	07–147	Ram	3	A	Sup.	Hybrid (F2)	0.50
	07–148	Ewe	2	?	-	Hybrid (F2)	0.89

1 Individuals were identified as of pure-supplemented lineage.

newhybrids was effective at detecting hybrid individuals using our molecular data. We calculated 1.1% and 0.7% overall error rates (i.e., overall rate of false negatives) for detecting hybrid individuals using simulated hybrids from the SM and LG supplementations, respectively (Table 4). However, accurate assignment of individuals to a particular category of hybrid descent degenerated from recent hybrids (i.e., F1) through later hybrid classes (Table 4). Thus, in our analyses we relied only on the distinction between residents and hybrids, regardless of the assignment to a particular category of hybrid descent.

**Table 4:** Assignments using simulated, known genotypes designed to assess the accuracy of program newhybrids to detect various categories of interpopulation hybrids (i.e., admixed individuals) using multilocus microsatellite data from Steens Mountain (SM) and Leslie Gulch (LG)

Population	Simulated genotypes	Assignment						Hybrid^1^	Hybrid error	Hyrbid class error
					
		Res.	Sup.	F_1_	F_2_	BX_res_	BX_sup_			
SM	F_1_	0	0	782	38	139	41	1000	0.0%	21.8%
	F_2_	0	4	241	343	222	190	996	0.4%	75.9%
	BX_res_	19	0	144	32	805	0	981	1.9%	85.6%
	BX_sup_	0	22	210	88	13	667	978	2.2%	79.0%
	Total	19	26	1377	501	1179	898	3955	1.1%	–
LG	F_1_	0	0	779	61	87	73	1000	0.0%	22.1%
	F_2_	0	0	268	375	149	208	1000	0.0%	62.5%
	BX_res_	14	0	191	62	730	3	986	1.4%	27.0%
	BX_sup_	0	12	151	82	4	751	988	1.2%	24.9%
	Total	14	12	1389	580	970	1035	3974	0.7%	–

1 The hybrid category was a sum of individuals assigned to F_1_, F_2_, and the two backcross categories. Res. = resident; Sup. = supplemented.

### Diagnosing the potential for outbreeding depression

**Figure 3:**
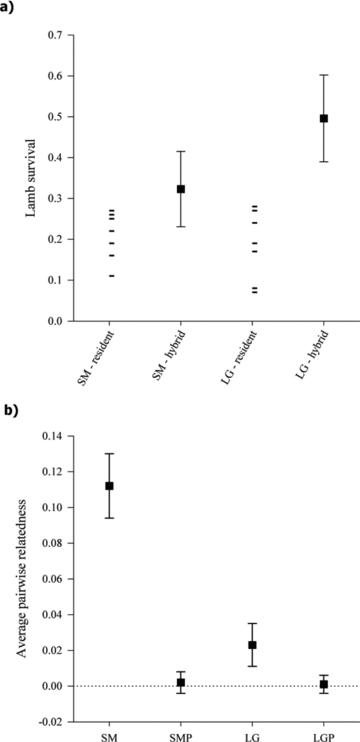
(a) Simulated (mean ± 2 standard deviation) lamb survival values for hybrid individuals were equal to or higher than baseline lamb survival values (bars) observed for Steens Mountain (SM) and Leslie Gulch (LG) resident populations prior to supplementation during 1990–1999. (b) Average pairwise relatedness decreased after experimental supplementation of two populations of bighorn sheep in Oregon, SM and LG. Postsupplementation levels are indicated as SM postsupplementation for SM and LG postsupplementation for LG. Experimental populations were sampled 6 years after supplementation in 2000 for SM and 2001 for LG to gauge the success of genetic management.

The stable age distribution (based on mean demographic parameters inTable 1) indicated 72% of each population would be ≤6 years of age, and thus available for postsupplementation sampling. Therefore, adjusted populations sizes were 126 for SMP (175 population size estimate × 0.72) and 180 for LGP (250 population size estimate × 0.72). The proportion of hybrids in SMP was 20.5% and at LGP was 23.4% (see *Documenting Introgression* above). We calculated the expected number of hybrid individuals as 25.7 in SMP (20.5%× adjusted population size of 126) to compare against the stochastic lamb survival models, or 23.1 to 28.3 hybrid individuals within ±10% of our expectation. Similarly, we calculated the expected number of hybrid individuals in LGP as 42.1 (23.4%× adjusted population size of 180) or 37.9 to 46.3 hybrid individuals within ±10% of our expectation. The average and central 95% of lamb survival values for simulations in which the number of hybrid individuals fell between ±10% calculated values for SMP did not fully extend beyond presupplementation baseline values, but generally was higher (Fig. 3a). Lamb survival values for LGP interpopulation hybrids exceeded presupplementation resident levels (Fig. 3a).

### Describing population-level effects of genetic management

H_E_ was not significantly higher in SMP (*t* = –1.67, df = 15, *P* = 0.115), but A_R_ did increase after genetic management (*t* = –3.90, df = 15, *P* = 0.001; Table 2). Both H_E_ (*t* = –2.44, df = 15, *P* = 0.028) and A_R_ increased in LGP (*t* = –4.61, df = 15, *P* < 0.001; Table 2). Similarly, average pair-wise relatedness decreased from levels significantly greater than 0 to nonsignificant levels of pairwise relatedness in both SMP and LGP (Fig. 3b). The number of mtDNA haplotypes increased from one prior to supplementation to three in both SMP and LGP (Table 2). Concomitant with the influx of new haplotypes, haplotype diversity (H_D_) and the average number of pairwise nucleotide differences among haplotypes within each population (*k*) increased beyond the presupplementation levels (i.e., >0) in both SMP and LGP (Table 2).

## Discussion

Supplementation of reintroduced populations, especially those suffering from the negative effects of inbreeding depression, can act as a powerful tool to increase genetic diversity and may enhance the probability of population persistence (Ahlroth et al. 2003; Van Houtan et al. 2009). Bighorn sheep populations are ideal candidates for genetic management programs that incorporate supplementations to achieve introgression. Fully half of the extant populations of bighorn sheep in North America derive from reintroduction efforts (Krausman 2000): efforts often introducing few founding individuals (e.g., <20 individuals; Ramey 1993) into habitat fragments with little possibility for genetic exchange via occasional natural migrants (Singer et al. 2001). Moreover, these populations are becoming increasingly isolated as a result of human development (Epps et al. 2005) and climate change (Epps et al. 2006). These relatively small and isolated populations suffer considerable risk of inbreeding depression (Ingvarsson 2001; Leberg and Firmin 2008). Thus, genetic management of reintroduced bighorn sheep populations may be critical for their long-term persistence.

While there is little doubt that population supplementation can alleviate small population problems associated with inbreeding depression, genetic introgression also may be associated with some potential risks (Tallmon et al. 2004). For instance, outbreeding depression can have consequences for life-history traits important for population persistence as severe as those caused by inbreeding depression (Marshall and Spalton 2000), and usually results from the disruption of coadapted gene complexes (Templeton 1986; Whitlock et al. 1995; Edmands 2007; Hedrick and Fredrickson 2010). Because outbreeding depression readily occurs in crosses between populations within a species (Edmands 1999, 2007), diagnosing the potential for outbreeding depression is critical for genetic management programs, especially when suitable populations are being identified as sources for translocations (Leberg 1993; Hedrick and Fredrickson 2010). Numerous recent studies provide theoretical (Ingvarsson and Whitlock 2000; Whitlock et al. 2000) and empirical (Spielman and Frankham 1992; Ball et al. 2000; Saccheri and Brakefield 2002; Hogg et al. 2006) evidence of the positive, population-level effects associated with introgression of immigrant genes when populations suffer from inbreeding depression (Tallmon et al. 2004). However, the potential for outbreeding depression rarely has been evaluated as part of management activities (Edmands 2007), particularly among wild populations (Frankham 2010).

We used pre- and posttreatment genetic and demographic data to evaluate the potential for outbreeding depression in the context of an experimental supplementation of bighorn sheep in Oregon. Postsupplementation sampling revealed relatively high proportions of hybrid individuals in Steens Mountain (SMP) and Leslie Gulch (LGP) approximately one generation after supplementation: 20.5% (9 of 44 ≤6 year-old individuals sampled) in SMP and 23.4% (11 of 47) in LGP. In fact, additional introgression seems probable based on the detection of a small number of pure-supplemented individuals in postsupplementation samples from both treatment populations. Further, simulations incorporating stochastic demographic parameters demonstrated that lamb survival—a life-history parameter often impacted by inbreeding and outbreeding depression (Edmands 2007)—was at least no different for interpopulation hybrids than for presupplementation resident individuals in SM, and was higher for hybrids than presupplementation, resident individuals in LG.

Because interpopulation hybrids from both experimental populations exhibited equal or greater lamb survival than lambs in SM and LG populations prior to supplementation, we found no evidence of immediate outbreeding depression. Moreover, our results could suggest that admixed individuals were more fit than residents indicating the possibility of a genetic rescue effect (Thrall et al. 1998). Outbreeding depression in first-generation hybrids can occur as a result of underdominance, epistatic interactions, or the disruption of local adaptations (Edmands 2007). Although we do not expect local adaptations to have evolved between the populations in our study, the possibility of underdominance or epistatic interactions remains. It should be noted that the effects of outbreeding depression can delay until F2 or later hybrid crosses (Templeton 1986; Leberg 1993; Edmands 2007). Unfortunately, assessing the fitness of later generation hybrids is challenging in a wild population exhibiting overlapping generations. For example, simulations based on our data revealed that we were unlikely to accurately assign individuals to a particular category of hybrid descent. However, those simulations also demonstrated that we had excellent power to distinguish hybrids in general from pure resident or pure supplemented individuals (seeTable 4).

At the population level, our analyses revealed a consistent signature of increased genetic diversity in both treatment populations postsupplementation (i.e., SMP and LGP). Specifically, sampling of SMP revealed 15% higher heterozygosity (though this difference was not statistically significant), 24% higher allelic richness, and increased mitochondrial haplotype diversity and, in keeping with our expectations, average pairwise relatedness decreased from significant levels in SM to nonsignificant levels in SMP. In the replicate population, LG, expected heterozygosity and allelic richness increased 12% and 23%, respectively. Similar to results in SM, average pairwise relatedness decreased in LGP and the number of mtDNA haplotypes and measures of mitochondrial diversity in LG increased after genetic management.

In conclusion, we found that genetic management in a replicated, experimental framework successfully increased genetic diversity in California bighorn sheep populations in Oregon by facilitating the introgression of immigrant genotypes. In addition, this study was unique in its progression through steps recently advocated byHedrick and Fredrickson (2010) for attempts at genetic rescue. Indicators of inbreeding depression were identified in wild populations, a suitable source population was identified for an attempt at genetic rescue, experimental supplementations were conducted (Whittaker et al. 2004), and, finally, the current work assesses the outcomes of those experimental supplementations. In particular, we developed a unique approach incorporating demographic models and genetic analyses to discern the ranges of juvenile survival achieved by interpopulation hybrids in the two populations, and in doing so, assessed the potential for outbreeding depression between the putative source and resident populations. Given no evidence for outbreeding depression as a result of the experimental supplementations, evidence of strong introgression between supplemented and resident individuals, and increases in genetic diversity in replicate populations, we suggest further genetic management of bighorn sheep in Oregon. In addition, this study may serve as a model to inform similar genetic management programs for reintroduced species elsewhere.

## Appendix 1

Workflow depicting the methods by which results from genetic analyses and stochastic demographic simulations were synthesized to determine probable values of lamb survival, a demographic parameter impacted by outbreeding depression, for interpopulation hybrids (i.e., resident × supplemented individuals) using data from an experiment in genetic management of California bighorn sheep in Oregon.

## Appendix 2

Microsatellite and mitochondrial markers used in this study. Primer sequences, optimized annealing temperatures (T_A_), concentrations of MgCl_2_, cycles in the PCR reaction, fragment size range, and the number of alleles detected using 415 California bighorn sheep sampled from a larger sample set including populations in Oregon, British Columbia, and Nevada (Z. Olson, unpublished data). Four additional loci, BM1818^1^, CELJP23^2^, OarAE16^14^, and TGLA94^4^ did not amplify reliably and were not further analyzed.

**Table:**

Marker	Primer sequences (5′→ 3′)	T_A_ (°C)	MgCl_2_ (mM)	Cycles	Size range (bp)	Alleles
Microsatellite							
BM1225^1^	F: tttctcaacagaggtgtccac	60	0.8	30	243–261	4
	R: acccctatcaccatgctctg					
BM203^1^	F: gggtgtgacattttgttccc	60	1.8	30	218–246	6
	R: ctgctcgccactagtccttc					
BM4107^1^	F: agcccctgctattgtgtgag	58	1.4	30	145	1
	R: ataggctttgcattgttcagg					
BM4505^1^	F: ttatcttggcttctgggtgc	63	1.8	30	253–277	9
	R: atcttcacttgggatgcagg					
BM6506^1^	F: gcacgtggtaaagagatggc	57	1.4	35	197–215	4
	R: agcaacttgagcatggcac					
BM848^1^	F: tggttggaaggaaaacttgg	52	0.8	30	219–243	6
	R: cctctgctcctcaagacac					
BMC1009^1^	F: gcaccagcagagaggacatt	58	1.4	30	276–284	3
	R: accggctattgtccatcttg					
BMC1222^5^	F: ccaattttgcagataagaaaaca	60	1.8	30	285–291	4
	R: cctgagtgttcctcctgagt					
CELB9^6^	F: tcaccttaatatggaggcagaaata	63	1.8	30	233–243	3
	R: gatgcatttcagattatggcttatc					
CELJP15^2^	F: ggaaataccttatctttcattcttgactgtgg	63	1.8	30	159–167	2
	R: ccttctttctcattgctaacttatattaaatatcc					
IRBP^3^	F: gtatgatcaccttctatgcttcc	60	1.8	30	168–198	4
	R: ccctaaatactaccatctagaag					
MAF209^7^	F: tcatgcacttaagtatgtaggatgctg	65	1.8	30	106–118	2
	R: gatcacaaaaagttggatacaaccgtgg					
MCM527^8^	F: gtccattgcctcaaatcaattc	50	0.8	30	157–171	5
	R: aaaccacttgactactccccaa					
OarCP26^9^	F: ggcctaacagaattcagatgatgttgc	67	0.8	30	128–148	6
	R: gtcaccatactgacggctggttcc					
OarFCB11^10^	F: ggcctgaactcacaagttgatatatctatcac	57	0.6	35	122–128	4
	R: gcaagcaggttctttaccactagcacc					
OarFCB193^10^	F: ttcatctcagactgggattcagaaaggc	55	0.8	30	102–118	4
	R: gcttggaaataaccctcctgcatccc					
OarFCB304^10^	F: ccctaggagctttcaataaagaatcgg	55	0.6	30	133–141	4
	R: cgctgctgtcaactgggtcaggg					
RT9^11^	F: tgaagtttaatttccactct	53	1.4	30	120–138	4
	R: cagtcactttcatcccacat					
TGLA126^4^	F: ctaatttagaatgagagaggcttct	53	0.8	35	114–138	7
	R: ttggtctctattctctgaatattcc					
Mitochondrial						
L15999/H16498^12^	F: accatcaacacccaaagctga	65	1.8			
	R: cctgaagtaggaaccagatg					
L15999/Alternate^13^	R: gtgagatggccctgaagaaaga	58	1.8			

^1^Marker described in Bishop et al. 1994, ^2^J. Pemberton unpublished data, ^3^Moore et al. 1992, ^4^Georges and Massey 1992, ^5^de Gortari et al. 1997, ^6^Tate 1997, ^7^Buchanan and Crawford 1992, ^8^Hulme et al. 1995, ^9^Ede et al. 1995, ^10^Buchanan and Crawford 1993, ^11^Wilson et al. 1997, ^12^Loehr et al. 2006, ^13^Alternate mtDNA primer was designed from our preliminary sequence data using primer3 software of Rozen and Skaletsky 2000, ^14^Penty et al. 1993.
